# Traffic‐related air pollution significantly aggravates the detrimental impact of infections on the risk of Alzheimer's disease, particularly in non‐carriers of APOE4

**DOI:** 10.1002/alz70860_104117

**Published:** 2025-12-23

**Authors:** Svetlana Ukraintseva, Vladimir Popov, Hongzhe Duan, Arseniy Yashkin, Igor Akushevich, Konstantin Arbeev, Anatoliy Yashin

**Affiliations:** ^1^ Social Science Research Institute, Duke University, Durham, NC, USA

## Abstract

**Background:**

Alzheimer's disease (AD) likely results from a combination of genetic, environmental, and other factors. Accumulating evidence points to a role of infections in AD. Traffic‐related air pollution (TRAP) and APOE4 are major risk factors for AD that may act together with infections to influence AD risk. Here, we investigated how the interplay between history of infections and exposure to TRAP impacts the risk of AD and other dementias (AD+) in carriers and non‐carriers of APOE4.

**Method:**

UK Biobank (UKB) participants aged 60‐75 years (51,069 females and 48,973 males) with information about ICD10 diagnoses and exposure to TRAP were included. High exposure to TRAP was approximated by the distance (50 meters or less) of a participant's residence to a major road. The rs429358 (C) allele represented APOE4 carrier status. Chi‐square test, Wilson score interval test, Wald interval test, Wald risk ratio test, Welch test, and regression were used to examine statistical significance.

**Result:**

UKB participants with a history of infections and exposure to TRAP had a 164% higher risk of AD+ (RR=2.64, 95% CI [1.79; 3.88], men and women combined), compared to individuals of the same age without either risk factor. Separately, infections without TRAP increased the risk of AD+ by 54% (RR=1.54, 95% CI [1.32; 1.78]). The impact of TRAP without infections on AD+ was not significant. In non‐carriers of APOE4 with a history of infections and exposure to TRAP, the relative risk of AD+ was 4.49 (95% CI [2.68; 7.50]) compared to subjects without either risk factor. In APOE4 carriers, associations between history of infections and exposure to TRAP with the risk of AD were not significant.

**Conclusion:**

Chronically high exposure to TRAP significantly aggravates the detrimental impact of infections on the risk of AD and other dementias in males and females aged 60‐75 years, particularly in non‐carriers of APOE4. Weaker associations in APOE4 carriers may be explained by APOE4 being a very strong AD risk factor, whose AD‐promoting effects may outweigh the effects of other risk factors.

